# Synthesis and Biological Evaluation of 3-Aryl-quinoxaline-2-carbonitrile 1,4-Di-*N*-oxide Derivatives as Hypoxic Selective Anti-tumor Agents

**DOI:** 10.3390/molecules17089683

**Published:** 2012-08-13

**Authors:** Yunzhen Hu, Qing Xia, Shihao Shangguan, Xiaowen Liu, Yongzhou Hu, Rong Sheng

**Affiliations:** 1 ZJU-ENS Joint Laboratory of Medicinal Chemistry, Zhejiang University, Hangzhou 310058, China; 2 Department of Pharmacy, the First Affiliated Hospital of college of Medicine, Zhejiang University, Hangzhou 310006, China; 3 Institute of Pharmacology & Toxicology, College of Pharmaceutical Sciences, Zhejiang University, Hangzhou 310058, China

**Keywords:** 3-aryl-2-quinoxaline-carbonitrile 1,4-dioxide, hypoxic cytotoxic activity, SAR

## Abstract

A series of 3-aryl-2-quinoxaline-carbonitrile 1,4-di-*N*-oxide derivatives were designed, synthesized and evaluated for hypoxic and normoxic cytotoxic activity against human SMMC-7721, K562, KB, A549 and PC-3 cell lines. Many of these new compounds displayed more potent hypoxic cytotoxic activity compared with TX-402 and TPZ in the tumor cells based evaluation, which confirmed our hypothesis that the replacement of the 3-amine with the substituted aryl ring of TX-402 increases the hypoxic anti-tumor activity. The preliminary SAR revealed that 3-chloro was a favorable substituent in the phenyl ring for hypoxic cytotoxicity and 7-methyl or 7-methoxy substituted derivatives exhibited better hypoxic selectivity against most of the tested cell lines. The most potent compound, 7-methyl-3-(3-chlorophenyl)-quinoxaline-2-carbonitrile 1,4-dioxide (**9h**) was selected for further anti-tumor evaluation and mechanistic study. It also exhibited significant cytotoxic activity against BEL-7402, HepG2, HL-60, NCI-H460, HCT-116 and CHP126 cell lines in hypoxia with IC_50_ values ranging from 0.31 to 3.16 μM, and preliminary mechanism study revealed that **9h** induced apoptosis in a caspase-dependent pathway.

## 1. Introduction

Hypoxia is an inevitable circumstance in most solid tumors resulting from rapid tumor growth with an inefficient microvascular system. Tumor cells within these regions show resistance to radiotherapy and chemotherapy and present a tremendous challenge to cancer therapy [1,2]. Hypoxia also distinguishes solid tumor cells from physiologically normal cells and is marked as an attractive and exploitable therapeutic target. Five classes of chemical moieties (quinones, nitroimidazoles, aromatic N-oxides, aliphatic N-oxides and transition metal complexes) have been identified as hypoxic cytotoxins in recent years. These compounds were selectively activated by reductive enzymes within hypoxic environment and generated toxic metabolites causing cell death [3].

**Figure 1:**
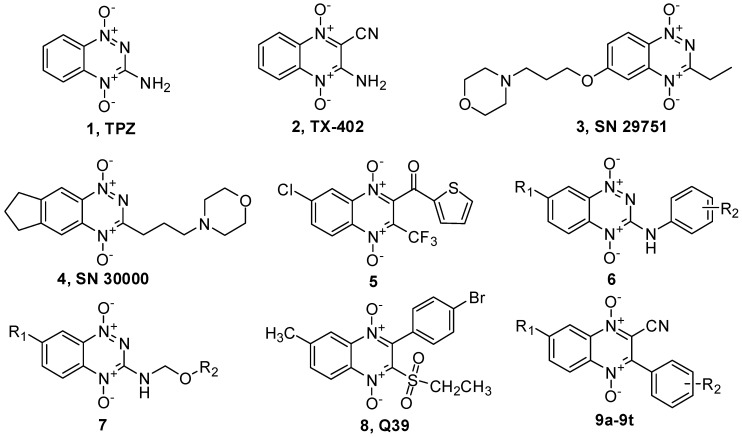
The structures of aromatic *N*-oxides with hypoxic cytotoxic activity.

As classical aromatic N-oxides derivatives, tirapazamine (3-aminobenzotriazine-1,4-dioxide, **1**, TPZ, Figure 1) and 3-amino-2-quinoxalinecarbonitrile 1,4-di-*N*-oxide (TX-402, **2**, Figure 1) were extensively studied in the past years. TPZ was bioreductively activated through the one-electron reduction of the benzotriazine-l,4-di-*N*-oxide moiety by reductase to form hydroxyl and benzotriazinyl radicals that cause DNA damage. It had already been introduced into phase II and III clinical trials in combination with radiotherapy and chemotherapy for advanced head and neck cancers [4,5]. TX-402 also exhibited efficient hypoxic selective anti-tumor activities against various tumor cells with a similar DNA damage mechanism [6]. Although both of them exhibited poor extravascular transport, the unique one-electron reduction activation mechanism and encouraging antitumor profiles have stimulated in recent years intense research efforts in the design and synthesis of a variety of TPZ and TX-402 derivatives [7,8,9,10]. For example, benzotriazine-1,4-dioxide derivatives SN 29751 (**3**, Figure 1) and SN 30000 (**4**, Figure 1) were identified as the promising secondary generation TPZ analogues by using a spatially resolved pharmacokinetic/pharmacodynamic (SR-PKPD) model that considers tissue penetration explicitly during lead optimization [7,10]. Beatriz reported the synthesis and the biological evaluation of a series of 2-arylcarbonyl-3-trifluoromethylquinoxaline-1,4-di-N-oxide derivatives. The most potent compound 2-(thiophene-2-carbonyl)-3-trifluoromethylquinoxaline 1,4-di-N-oxide (**5**, Figure 1) not only exhibited good cytotoxic activity against NCI 60 cell lines with mean GI_50_ value of 0.07 μM, but also showed positive activity in an *in vivo* hollow fiber assay [8].

To overcome the poor extravascular transport of TPZ, our lab have synthesized and evaluated 3-aryl amino and 3-(alkoxymethylamino) benzotriazine-1,4-dioxide derivatives **6** and **7** (Figure 1) through introduction of lipophilic groups into the C-3 amino of TPZ. Most of these compounds were more potent than TPZ in the tumor cell lines assay and some of them exhibited higher hypoxia selectivity. The preliminary SAR study revealed that the introduction of an aromatic group at the C-3 amino was favorable for hypoxic cytotoxic activity and the physico-chemical study showed a positive correlation between hypoxic activity and lipophilicity within a certain range [11,12,13].

With a similar drug design strategy, we also synthesized a series of 3-phenyl-2-ethylthio- (or 2-ethylsulfonyl)-quinoxaline-1,4-dioxide derivatives through replacement of the 2-cyano and 3-amine moieties with 2-ethylthio (or 2-ethysulfonyl) and the 3-aryl of TX-402. The 2-ethylsulfonyl derivatives displayed moderate to good antiproliferative activity in hypoxia, while the 2-ethylthio derivatives showed almost no activity in the cell-based test. These results implicated that the electron-withdrawing 2-ethylsulfonyl moiety was necessary for hypoxic activity probably due to its modulation of the one-electron reduction potential of molecules. Among all the synthesized compounds, 3-(4-bromophenyl)-2-(ethylsulfonyl)-6-methylquinoxaline-1,4-dioxide (Q39, **8**, Figure 1) not only exhibited good antiproliferative activity in extensive cell lines in hypoxia, but also inhibited SMMC-7721 tumor growth in a dose-dependent manner in a human tumor xenograft mice model [14,15,16].

Based on these research results, we have envisioned that replacement of the 3-amino moiety of TX-402 with a substituted aryl would be favorable for hypoxic anti-tumor activity. To find new lead compounds with enhanced potency and hypoxic selectivity, we report here the design, synthesis and evaluation of a series of 3-aryl-2-quinoxalinecarbonitrile-1,4-di-*N*-oxide derivatives **9a**–**t** (Figure 1) as hypoxic selective anti-tumor agents. Although compounds containing this skeleton have been reported as antimalarial agents [17,18,19], their hypoxic anti-tumor characteristic has never been disclosed. The main objective of present study was to investigate the effect of the replacement of the 3-amine moiety with a substituted 3-aryl moiety and the modification of substituent at the 7-position of TX-402 on anti-tumor activity and hypoxic selectivity. The study also has led to the identification of several new potent hypoxic selective anti-tumor compounds.

## 2. Results and Discussion

### 2.1. Chemistry

**Scheme 1:**
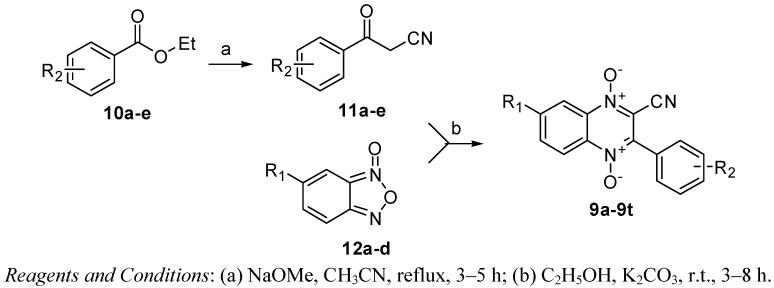
The synthetic route to compounds **9a**–**t**.

The synthetic route of 3-aryl-2-quinoxalinecarbonitrile-1,4-di-*N*-oxides **9a**–**t** is shown in Scheme 1. Refluxing of substituted benzoates **10a**–**e** with acetonitrile in the presence of sodium methoxide provided arylacetonitriles **11a**–**e**, followed by the classical Beirut reaction with 5-substituted benzofuroxans **12a**–**d** in ethanol with catalytic amount of potassium carbonate at room temperature to yield target compounds **9a**–**t**. The structures of all the newly synthesized compounds were confirmed by IR, ^1^H-NMR and HRMS.

### 2.2. Pharmacology

#### 2.2.1. *In Vitro* Cytotoxic Activity

All the newly synthesized compounds were assayed for *in vitro *cytotoxicity against five human cancer cell lines, including SMMC-7721 (hepatoma), K562 (chronic myeloid leukemia), KB (epidermoid carcinoma of the nasopharynx), A549 (nonsmall cell lung carcinoma) and PC-3 (prostate cancer) under normoxic and hypoxic conditions. TX-402 and TPZ were employed as positive controls and the antiproliferative activity results are summarized in Table 1.

As shown in Table 1, many of 3-aryl-2-quinoxalinecarbonitrile-1,4-di-*N*-oxide derivatives showed higher or similar hypoxic cytotoxic activity and selectivity in comparison with those of TX-402 and TPZ against most of the tested cell lines, in particular for the SMMC-7721, K562 and KB cell lines.

Obviously, the hypoxic cytotoxic potency of **9a**–**t** was highly dependent on the 3-position and 7-position substitutents of the quinoxaline. Such as, compound **9a** (3-phenylquinoxaline-2-carbonitrile 1,4-dioxide) exhibited weak to good cytotoxicity against the SMMC-7721, K562, KB, A549 and PC3 cell lines (IC_50_ = 1.58, 17.53, 1.53, 8.08 and 25.0 μM, respectively). Compound **9h** (7-methyl-3-(3-chlorophenyl)-quinoxaline-2-carbonitrile 1,4-dioxide) showed good hypoxic cytotoxic activity against five cell lines, with IC_50_ values of 0.76, 0.92, 0.53, 4.91 and 2.25 μM, respectively. By comparison with the IC_50_ values of the TX-402 (>50, 13.1, 0.98, >50 and 5.87 μM, respectively), our hypothesis that the replacement of 3-amine with substituted aryl ring of TX-402 increased the hypoxic anti-tumor activity was confirmed.

The substituents on the 3-phenyl moiety affect the anti-tumor activity by changing the electronic and lipophilic properties of the entire molecule. Comparing the cytotoxic activity of **9c** and **9h** with that of **9a** and **9f** suggested that an electron-withdrawing 3-chloro group in the 3-phenyl moiety increased cytotoxicity against most tested cell lines, particularly for the SMMC-7721, K562 and KB cell lines. The substituents on the 7-position of the quinoxaline ring also have a significant impact on anti-tumor activity and hypoxia selectivity because of the disparity in the electronic properties of the resulting molecules. As shown in Table 1, 7-chloro derivatives **9p** and **9q** exhibited better hypoxic antiproliferative activity than the 7-unsubstituted derivative **9a** and **9b** in most tested cell lines.

**Table 1:** Cytotoxicity of 2-cyano-3-aryl-quinoxaline 1,4-dioxides against five cancer cell lines in hypoxia and in normoxia.

Comp.	R_1_	R_2_	Cytotoxicity(IC_50_, μM) and HCR														
			SMMC-7721			K562			KB			A549			PC3		
			H ^a^	N ^b^	HCR ^c^	H	N	HCR	H	N	HCR	H	N	HCR	H	N	HCR
TX-402	-	-	>50	>50	-	13.1	>50	-	0.98	8.85	9.03	>50	>50	-	5.87	>50	-
TPZ	-	-	4.75	32.79	6.90	1.81	19.41	10.72	18.71	6.29	0.34	1.93	7.43	3.85	1.17	20.97	17.92
**9a**	H	H	1.58	15.7	9.94	17.53	45.4	2.59	1.53	16.46	10.76	8.08	52	6.44	25	24.6	0.98
**9b**	H	3-CH_3_	5.07	100	19.72	17.7	15.7	0.89	7.9	14.75	1.87	21	26.3	1.25	18.2	7.56	0.42
**9c**	H	3-Cl	1.09	5	4.59	1.03	11.32	10.99	0.76	4.59	6.04	10.61	46.71	4.40	3	15.03	5.01
**9d**	H	4-Br	1.82	32.9	18.08	19.58	47.1	2.41	5.08	13.61	2.68	11.2	19.8	1.77	16.4	>50	>3.05
**9e**	H	4-NO_2_	1.43	8.28	5.79	4.01	3.98	0.99	2.83	12	4.24	8.02	42.31	5.28	10.36	>50	>4.83
**9f**	CH_3_	H	0.63	100	158.73	2.04	22.6	11.08	4.54	34.9	7.69	9.38	12.5	1.33	3.8	24.2	6.37
**9g**	CH_3_	3-CH_3_	2.98	2.97	1.00	0.64	3.16	4.94	0.72	10.65	14.79	21.72	>50	>2.30	6.21	>50	>8.05
**9h**	CH_3_	3-Cl	0.76	10.34	13.61	0.92	7.54	8.20	0.53	2.19	4.13	4.91	11.13	2.27	2.25	12.55	5.57
**9i**	CH_3_	4-Br	0.93	6.18	6.65	1.07	4.32	4.04	0.17	5.96	35.06	36.15	32.46	0.90	5.54	20.26	3.66
**9j**	CH_3_	4-NO_2_	1.6	43	26.88	6.59	13.3	2.02	5.12	8.71	1.70	17	25	1.47	28.3	39	1.38
**9k**	OCH_3_	H	1.16	78	67.24	7.98	16.8	2.11	3.97	66.72	16.81	6.31	4.46	0.71	6.76	43.5	6.43
**9l**	OCH_3_	3-CH_3_	1.7	6.23	3.66	0.76	13.53	17.80	1.16	6.23	5.37	15.3	>50	>3.27	4.41	>50	>11.34
**9m**	OCH_3_	3-Cl	0.9	4.83	5.37	2.02	6.76	3.35	1.42	4.87	3.43	8.75	>50	>5.71	2.25	17.55	7.80
**9n**	OCH_3_	4-Br	0.12	13.84	115.33	1.37	3.34	2.44	4.03	6.63	1.65	11.32	28.43	2.51	8.4	28.61	3.41
**9o**	OCH_3_	4-NO_2_	1.62	3.92	2.42	4.49	9.23	2.06	5.41	3.54	0.65	6.17	6.53	1.06	18.7	25.2	1.35
**9p**	Cl	H	0.46	4.27	9.28	2.66	9.39	3.53	0.33	3.25	9.85	5.75	30.84	5.36	3.89	27.19	6.99
**9q**	Cl	3-CH_3_	0.37	1.73	4.68	0.79	0.87	1.10	0.62	11.85	19.11	12.3	18.5	1.50	9.93	>50	>5.04
**9r**	Cl	3-Cl	0.8	1.73	2.16	1.73	4.62	2.67	0.51	1.92	3.76	11.77	11.74	1.00	3.06	11.98	3.92
**9s**	Cl	4-Br	2.08	23.7	11.39	16.55	38.5	2.33	3.63	17.82	4.91	33.59	4.1	0.12	50.9	13.3	0.26
**9t**	Cl	4-NO_2_	3.4	23.6	6.94	1.67	1.48	0.89	4.92	2.38	0.48	5.72	5.4	0.94	16.1	13.8	0.86

^a^ H = Hypoxia: 3% percentage of oxygen. ^b^ N = Normoxia: 20% percentage of oxygen. ^c^ HCR, hypoxic cytotoxicity ratio.

On the other hand, the introduction of electron-donating methyl or methoxy groups into the 7-position of the quinoxaline ring improved the hypoxic selectivity against most cell lines, in particular for the SMMC-7721, K562 and KB cell lines. For example, 7-methyl and 7-methoxy-substituted quinoxaline derivatives **9f** and **9n** showed very high hypoxic selectivity against SMMC-7721 cell line, with HCR values of 159 and 115, respectively, which were 23- and 16.7-fold more selective than TPZ (HCR = 6.90). The 7-methyl-substituted quinoxaline derivative **9i** was the most hypoxic selective cytotoxin against the KB cell line (HCR value of 35.1), which is an 11.8-fold improvement compared with TPZ (HCR value of 2.97).

Among all the five tested cell lines, the SMMC-7721 was the most sensitive cell line to these newly synthesized quinoxaline derivatives, with IC_50_ values in the 0.37–5.07 μM range and HCR values between 1.0 and 158.73. The A549 one was the most resistant cell line to the hypoxic cytotoxic effect of these derivatives, with IC_50_ values in the range of 5.72–36.15μM and HCR values between 0.12 and 5.71. This result was consistent with that of 2-arylcarbonyl-3-trifluoromethylquinoxaline-1,4-di-*N*-oxide derivatives [8], suggesting that these two series of quinoxaline-1,4-di-*N*-oxide derivatives may possess similar anti-tumor characteristics.

Compound **9h** aroused our great interest because of its high hypoxic antiproliferative activity against all the five cell lines with IC_50 _values range from 0.53 to 4.91 μM. It was further evaluated in other six tumor cell lines in hypoxia and in normoxia, including Human hepatoma BEL-7402, HepG2, Human promyelocytic leukemia HL-60, Human lung cancer NCI-H460, Human colon cancer HCT-116 and Human neuroblastoma CHP126. The results in Table 2 showed that **9h** also exhibited significant cytotoxicity against all six tested human tumor cell lines with IC_50_ values in the range of 0.31–3.16 μM in hypoxia. It also showed moderate to good hypoxia selectivity with HCR values between 1.52 and 17.8. These results suggest that **9h** might be a promising candidate for further development as hypoxic selective anti-tumor agent.

**Table 2:** Cytotoxic activity of **9h** against six human cancer cell lines in hypoxia and in normoxia.

Cell line	IC_50_ (mM)		HCR ^c^
	H ^a^	N ^b^	
Human hepatoma BEL-7402	2.23	14.7	6.60
Human hepatoma HepG2	1.76	13.1	7.44
Human promyelocytic leukemia HL-60	3.16	4.80	1.52
Human lung cancer NCI-H460	2.64	5.96	2.26
Human colon cancer HCT-116	1.71	4.92	2.88
Human neuroblastoma CHP126	0.31	5.51	17.8

^a^ H = Hypoxia: 3% percentage of oxygen. ^b^ N = Normoxia: 20% percentage of oxygen. ^c^ HCR, hypoxic cytotoxicity ratio.

#### 2.2.2. Mechanism Studies

To investigate the mechanism of these newly synthesized quinoxaline derivatives, compound **9h** was further assayed for its effect on cell cycle progression and apoptosis-associated protein expression.

**Figure 2:**
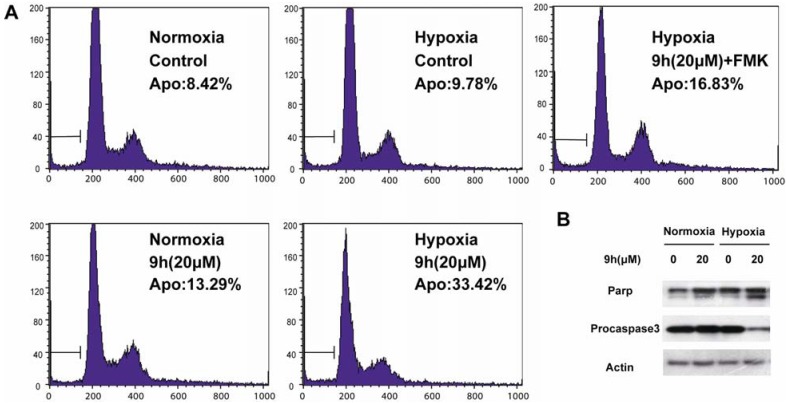
Pharmacological mechanism study of **9h**. (**A**) SMMC-7721 cells were incubated in normoxia and in hypoxia, and were treated with 9h (10 μM) for 48 h. After treatment, cells were harvested and detected of apoptosis by flow cytometry using PI apoptosis detection kit. (**B**) SMMC-7721cells were harvested after the same treatment as A, cell extract were collected and immunoblotted with procaspase-3 and PARP antibodies.

As shown in Figure 2A, spontaneous apoptosis (control) was seen in 8.42% of SMMC-7721 cells in normoxia and 9.78% in hypoxia. In normoxia, **9h** (20 μM) did not induce obvious apoptosis (13.4%) relative to controls. However, in hypoxia, it caused apoptosis in 33.42% of SMMC-7721 cells at 48 h. These data clearly demonstrated that **9h** exhibited a hypoxic-selective anti-tumor activity. Given that caspase signaling plays a critical role in stress induced apoptosis, we were thus encouraged to explore its role in **9h**-induced SMMC-7721 cells apoptosis. As illustrated in Figure 2A when SMMC-7721 cells were pretreated with pan-caspase inhibitor z-VAD-fmk (10.0 μM), **9h**-induced apoptosis was significantly reduced from 33.42% to 16.83% at 48 h (Figure 2A Collectively, these results indicated that **9h** serving as a potential hypoxic-selective compound and inducing apoptosis in a caspase-dependent pathway. In order to further validate our results, some proteins related to activation of caspase cascade were also detected. The expression of procaspase-3, and PARP and actin were measured in SMMC-7721 cells treated with **9h** (20.0 μM, 48 h). As shown in Figure 2B, **9h** decreased the protein levels of procaspase-3, and induce the cleavage of PARP in hypoxia. All these data further demonstrate the apoptosis triggered by **9h** in hypoxia is mediated by caspase signaling.

## 3. Experimental

### 3.1. General

Melting points were obtained on a B-540 Büchi melting-point apparatus and are uncorrected. IR spectra were performed on a Brüker VECTOR 22 FTIR spectrophotometer in KBr pellets (400–4000 cm^−1^). ^1^H-NMR spectra were recorded on a Brüker AM 500 instrument at 500 MHz (chemical shifts are expressed as δ values relative to TMS as internal standard). Mass spectra (MS), ESI (positive) were recorded on an Esquire-LC-00075 spectrometer. HRMS spectra were measured with an Agilent 6224 TOF LC/MS.

### 3.2. Chemistry

#### 3.2.1. General Procedure for the Synthesis of Benzoylacetonitriles **11a**–**e**

A mixture of ethyl benzoate **10a**–**e** (11.7 mmol), sodium methoxide (1.08 g, 20 mmol) and acetonitrile (15 mL) was refluxed for 3 h. After cooling to room temperature, the formed white precipitate was filtered and dissolved in water (50 mL). Three mol/L HCl (10 mL) was added to the solution and the mixture was extracted with CH_2_Cl_2_ (50 mL × 2). The combined organic layer was washed with brine and dried over anhydrous Na_2_SO_4_. The solvent was removed under reduced pressure to give the crude product, which was recrystallized from CH_2_C1_2_-petroleum ether to provide pure benzoylacetonitrile.

*Benzoylacetonitrile *(**11a**) [20]. White solid (83.1%), m.p.: 79–80 °C (lit. 80–81°C); ESI-MS: *m/z* = 146.3 [M + H]^+^.

*3-Methybenzoylacetonitrile *(**11b**) [21]. White solid (82.9%), m.p.: 74–75 °C (lit. 74–75 °C); ESI-MS: *m/z* = 160.6 [M + H]^+^. 

*3-Chlorobenzoylacetonitrile *(**11c**) [22]. White solid (81.6%), m.p.: 72–73 °C (lit. 71–73 °C); ESI-MS: *m/z* = 180.3 [M + H]^+^.

*4-Bromobenzoylacetonitrile *(**11d**) [23]. White solid (80.3%), m.p.: 160–161 °C (lit. 161–162 °C); ESI-MS: *m/z* = 225.2 [M + H]^+^.

*4-Nitrobenzoylacetonitrile *(**11e**) [23]. White solid (78.6%), m.p.: 122–123 °C (lit. 121–122 °C); ESI-MS: *m/z* = 191.3 [M + H]^+^.

#### 3.2.2. General Procedure for the Synthesis of 3-Aryl-2-quinoxalinecarbonitrile-1,4-di-*N*-oxide Derivatives **9a**–**t**

The substituted benzofuroxans **12a**–**d** were synthesized according to a literature method and confirmed by melting point comparison [24]. To a solution of benzoylacetonitrile **11a**–**e** (5.0 mmol) and benzofuroxan **12a**–**d** (5.0 mmol) in ethanol (40 mL), a 1% amount of potassium carbonate was added and the mixture was stirred at room temperature for 3 h. The precipitate was filtered and washed with ethanol to give a yellow solid, followed by recrystallization from ethanol to yield pure product.

*3-Phenylquinoxaline-2-carbonitrile-1,4-dioxide *(**9a**) [17]. Yellow solid (46.5%), m.p.: 208–210 °C; (lit. 206–207 °C) IR (KBr): ν 3092, 2235, 1625, 1594, 1490, 1343, 1090, 971, 770 cm^−1^; ^1^H-NMR (CDCl_3_) δ 8.69 (dd, 1H, *J_1_* = 9.0 Hz, *J_2_* = 1.0 Hz, H-5), 8.62 (dd, 1H, *J_1_* = 9.0 Hz, *J_2_* = 1.0 Hz, H-8), 7.99 (td, 1H, *J_1_* = 7.8 Hz, *J_2_* = 1.5 Hz, H-6), 7.94 (td, 1H, *J_1_* = 7.8 Hz, *J_2_* = 1.5 Hz, H-7), 7.72–7.74 (m, 2H, H-3′ and H-5′), 7.60–7.63 (m, 3H, H-2′, H-4′ and H-6′); ESI-MS: *m/z* = 264 [M + H]^+^.

*3-(3-Methylphenyl)quinoxaline-2-carbonitrile-1,4-dioxide *(**9b**). Yellow solid (48.0%); m.p.: 208–209 °C; IR (KBr): ν 3101, 2235, 1634, 1493, 1340, 1276, 773 cm^−1^; ^1^H-NMR (CDCl_3_) δ 8.68 (dd, 1H, *J_1 _* = 8.5 Hz, *J_2_* = 1.0 Hz, H-5), 8.61 (dd, 1H, *J_1_* = 8.5 Hz, *J_2_* = 1.0 Hz, H-8), 7.99 (td, 1H, *J_1_* = 8.0 Hz, *J_2_* = 1.5 Hz, H-6), 7.93 (td, 1H, *J_1_* = 8.0 Hz, *J_2_* = 1.5 Hz, H-7), 7.49–7.53 (m, 3H, H-4′, H-5′ and H-6′), 7.41–7.43 (m, 1H, H-2´), 2.46 (s, 3H, CH_3_); HRMS (TOF) calc. for C_16_H_12_N_3_O_2_ [M + H]^+^: 278.0924, found: 278.0927.

*3-(3-Chlorophenyl)quinoxaline-2-carbonitrile-1,4-dioxide *(**9c**). Yellow solid (53.3%); m.p.: 195–197 °C; IR (KBr): ν 3085, 2232, 1647, 1593, 1490, 1436, 1336, 1086, 976, 773 cm^−1^; ^1^H-NMR (CDCl_3_) δ 8.67 (d, 1H, *J* = 8.4 Hz, H-5), 8.61 (d, 1H, *J* = 8.4 Hz, H-8), 7.94–8.03 (m, 2H, H-6 and H-7), 7.75 (s, 1H, H-2′), 7.55–7.61 (m, 3H, H-4′, H-5′ and H-6′); HRMS (TOF) calc. for C_15_H_9_ClN_3_O_2_ [M + H]^+^: 298.0378, found: 298.0377.

*3-(4-Bromophenyl)quinoxaline-2-carbonitrile-1,4-dioxide *(**9d**). Yellow solid (47.2%); m.p*.*: 230–232 °C; IR (KBr): ν 3102, 2239, 1601, 1515, 1488, 1341, 1272, 1089, 978, 830, 772 cm^−1^; ^1^H-NMR (DMSO-d_6_) δ 8.51–8.55 (m, 2H, H-5 and H-8), 8.07–8.12 (m, 2H, H-6 and H-7), 7.80–7.83 (m, 2H, H-3′ and H-5′), 7.46–7.49 (m, 2H, H-2′ and H-6′); HRMS (TOF) calc. for C_15_H_9_BrN_3_O_2_ [M + H]^+^: 341.9873, found: 341.9868.

*3-(4-Nitrophenyl)quinoxaline-2-carbonitrile-1,4-dioxide *(**9e**). Yellow solid (48.5% yield); m.p.: 240–241 °C; IR (KBr): ν 3105, 2238, 1598, 1515, 1344, 1275, 1089, 979, 855 cm^−1^; ^1^H-NMR (DMSO-d_6_) δ 8.54–8.56 (m, 2H, H-5 and H-8), 8.47–8.49 (m, 1H, H-3′ and H-5′), 8.11–8.15 (m, 2H, H-6 and H-7), 8.03–8.05 (m, 2H, H-2′ and H-6′); HRMS (TOF) calc. for C_15_H_9_N_4_O_4_ [M + H]^+^: 309.0618, found: 309.0621.

*7-Methyl-3-phenylquinoxaline-2-carbonitrile-1,4-dioxide *(**9f**). [17]. Yellow solid (48.3% yield); m.p.: 191–193 °C (lit. 190–191 °C); IR (KBr): ν 3089, 2237, 1650, 1612, 1332, 1276, 1093, 831, 699 cm^–1^; ^1^H-NMR (CDCl_3_) δ 8.48–8.58 (m, 1H, H-5), 8.41 (s, 1H, H-8), 7.79–7.81 (m, 1H, H-6), 7.71–7.73 (m, 2H, H-3′ and H-5′), 7.62 (m, 3H, H-2′, H-4′ and H-6′), 2.67 (s, 3H, CH_3_); ESI-MS: *m/z* = 278.4 [M + H]^+^.

*7-Methyl-3-(3-methylphenyl)quinoxaline-2-carbonitrile-1,4-dioxide *(**9g**). Yellow solid (43.3% yield); m.p.: 216–217 °C; IR (KBr): ν 3109, 2236, 1613, 1484, 1328, 1275, 1091, 983, 828, 785 cm^−1^; ^1^H-NMR (CDCl_3_) δ 8.40–8.58 (m, 2H, H-5 and H-8), 7.77–7.80 (m, 1H, H-6), 7.49–7.52 (m, 3H, H-4′, H-5′ and H-6′), 7.42–7.43 (m, 1H, H-2′), 2.67 (s, 3H, CH_3_), 2.47 (s, 3H, CH_3_); HRMS (TOF) calc. for C_17_H_14_N_3_O_2_ [M + H]^+^: 292.1080, found: 292.1083.

*7-Methyl-3-(3-chlorophenyl)quinoxaline-2-carbonitrile-1,4-dioxide *(**9h**). Yellow solid (46.5% yield); m.p.: 221–222 °C; IR (KBr): ν 3096, 2233, 1593, 1491, 1333, 1088, 982, 830 cm^−1^; ^1^H-NMR (CDCl_3_) δ 8.50–8.57 (m, 1H, H-5), 8.40 (s, 1H, H-8), 7.80–7.82 (m, 1H, H-6), 7.75 (s, 1H, H-2′), 7.55–7.60 (m, 3H, H-4′, H-5′ and H-6′), 2.67 (s, 3H, CH_3_); HRMS (TOF) calc. for C_16_H_11_ClN_3_O_2_ [M + Na]^+^: 334.0354, found: 334.0352.

*7-Methyl-3-(4-bromophenyl)quinoxaline-2-carbonitrile-1,4-dioxide *(**9i**). Yellow solid (48.7%); m.p.: 222–223 °C; IR (KBr): ν 3076, 2239, 1602, 1501, 1330, 1231, 1088, 982, 836 cm^−1^; ^1^H-NMR (CDCl_3_, 500 MHz) δ 8.39–8.56 (m, 2H, H-5 and H-8), 7.80 (dd, 1H, *J_1_* = 8.5 Hz, *J_2_* = 1.0 Hz, H-6), 7.74–7.77 (m, 2H, H-3′ and H-5′), 7.28–7.31 (m, 2H, H-2′ and H-6′), 2.67 (s, 3H, CH_3_); HRMS (TOF) calc. for C_16_H_11_BrN_3_O_2_ [M + H]^+^: 356.0029, found: 356.0032.

*7-Methyl-3-(4-nitrophenyl)quinoxaline-2-carbonitrile-1,4-dioxide *(**9j**). Yellow solid (46.2%); m.p.: 240–241 °C; IR (KBr): ν 3106, 2237, 1602, 1519, 1341, 1092, 981, 936, 828 cm^−1^; ^1^H-NMR (CDCl_3_, 500 MHz) δ 8.52–8.58 (m, 1H, H-5), 8.46 (d, 2H, *J* = 8.5 Hz, H-3′ and H-5′), 8.43 (s, 1H, H-8), 7.95 (d, 2H, *J* = 8.5 Hz, H-2′ and H-6′), 7.82–7.85 (m, 1H, H-6), 2.69 (s, 3H, CH_3_); HRMS (TOF) calc. for C_16_H_10_N_4_NaO_4 _[M + Na]^+^: 345.0594, found: 345.0597.

*7-Methoxy-3-phenylquinoxaline-2-carbonitrile 1,4-dioxide *(**9k**). [17]. Yellow solid (45.1%); m.p.: 217–219 °C, (lit. 222–223 °C); IR (KBr): ν 3097, 2239, 1610, 1496, 1332, 1249, 1091, 845, 753 cm^−1^; ^1^H-NMR (CDCl_3_, 500 MHz) δ 8.58 (d, 1H, J = 9.6 Hz, H-5), 7.88 (d, 1H, *J* = 2.8 Hz, H-8), 7.70–7.72 (m, 2H, H-3′ and H-5′), 7.60–7.61 (m, 3H, H-2′, H-4′ and H-6′), 7.55 (dd, 1H, *J_1_* = 9.6 Hz, *J_2_* = 2.8 Hz, H-6), 4.06 (s, 3H, OCH_3_); ESI-MS: *m/z* = 294.2 [M + H]^+^.

*7-Methoxy-3-(3-methylphenyl)quinoxaline-2-carbonitrile 1,4-dioxide *(**9l**). Yellow solid (44.5%); m.p.: 203–204 °C; IR (KBr): ν 3101, 2235, 1610, 1504, 1395, 1329, 1258, 1131, 1012, 945, 849 cm^−1^; ^1^H-NMR (DMSO-d_6_, 500 MHz) δ 8.43 (d, 1H, *J* = 9.0 Hz, H-5), 7.79 (d, 1H, *J* = 3.0 Hz, H-8), 7.71 (dd, 1H, *J_1_* = 9.0 Hz, *J_2_* = 3.0 Hz, H-6), 7.47–7.53 (m, 3H, H-4′, H-5′ and H-6′), 7.41–7.43 (m, 1H, H-2′), 4.03 (s, 3H, OCH_3_), 2.40 (s, 3H, CH_3_); HRMS (TOF) calc. for C_17_H_14_N_3_O_3_ [M + H]^+^: 308.1030, found: 308.1033

*7-Methoxy-3-(3-chlorophenyl)quinoxaline-2-carbonitrile-1,4-dioxide *(**9m**). Yellow solid (45.7%); m.p.: 160–162 °C; IR (KBr): ν 3106, 2238, 1616, 1591, 1535, 1494, 1422, 1333, 1250, 1001, 814 cm^−1^; ^1^H-NMR (CDCl_3_) δ 8.56 (d, 1H, *J* = 9.6 Hz, H-5), 7.87 (d, 1H, *J* = 2.4 Hz, H-8), 7.74 (s, 1H, H-2′), 7.54–7.59 (m, 4H, H-6, H-4′, H-5′ and H-6′), 4.06 (s, 3H, CH_3_); HRMS (TOF) calc. for C_16_H_11_ClN_3_O_3_ [M + H]^+^: 328.0483, found: 328.0485.

*7-Methoxy-3-(4-bromophenyl)quinoxaline-2-carbonitrile-1,4-dioxide *(**9n**). Yellow solid (45.3%); m.p.: 184–186 °C; IR (KBr): ν 3092, 2238, 1608, 1504, 1332, 1248, 1016, 933, 836 cm^−1^; ^1^H-NMR (CDCl_3_) δ 8.56 (d, 1H, *J* = 9.5 Hz, H-8), 7.86 (d, 1H, *J* = 2.5 Hz, H-5), 7.73–7.76 (m, 2H, H-3′ and H-5′), 7.55 (dd, 1H, *J_1_* = 9.5 Hz, *J_2_* = 3.0 Hz, H-6), 7.27–7.31 (m, 2H, H-2′ and H-6′), 4.05 (s, 3H, OCH_3_); HRMS (TOF) calc. for C_16_H_11_BrN_3_O_3_ [M + H]^+^: 371.9978, found: 371.9979.

*7-Methoxy-3-(4-nitrophenyl)quinoxaline-2-carbonitrile-1,4-dioxide *(**9o**). Yellow solid (45.6%); m.p.: 238–240 °C; IR (KBr): ν 3105, 2235, 1615, 1525, 1361, 1327, 1255, 1013, 939, 858 cm^−1^; ^1^H-NMR (CDCl_3_) δ 8.57 (d, 1H, *J* = 9.0 Hz, H-5), 8.59 (d, 2H, *J* = 9.0 Hz, H-3′ and H-5′), 7.95 (d, 2H, *J* = 9.0 Hz, H-2′ and H-6′) , 7.90 (d, 1H, *J* = 2.5 Hz, H-8), 7.59 (dd, 1H, *J_1_* = 9.0 Hz, *J_2_* = 2.5 Hz, H-6), 4.08 (s, 3H, OCH_3_); HRMS (TOF) calc. for C_16_H_11_N_4_O_5_ [M + H]^+^: 339.0724, found: 339.0729.

*7-Chloro-3-phenylquinoxaline-2-carbonitrile-1,4-dioxide *(**9p**). [17]*.* Yellow solid (47.6%); m.p.: 221–223 °C (lit. 224–225 °C); IR (KBr): ν 3095, 2238, 1599, 1488, 1333, 1260, 1092, 984, 842, 770 cm^−1^; ^1^H-NMR (DMSO-d_6_) δ 8.55 (d, 1H, *J* = 9.0 Hz, H-5), 8.53 (s, 1H, H-8), 8.15 (dd, 1H, *J_1_* = 9.0 Hz, J_2_ = 2.0 Hz, H-6), 7.72–7.73 (m, 2H, H-3′ and H-5′), 7.61–7.63 (m, 3H, H-2′, H-4′ and H-6′); ESI-MS: *m/z* = 298.4 [M + H]^+^.

*7-Chloro-3-(3-methylphenyl)quinoxaline-2-carbonitrile-1,4-dioxide *(**9q**). Yellow solid (46.0%); m.p.: 215–216 °C; IR (KBr): ν 3099, 2235, 1636, 1489, 1400, 1325, 1260, 1095, 893, 831, 787 cm^−1^; ^1^H-NMR (DMSO-d_6_) δ 8.51–8.53 (m, 2H, H-5 and H-8), 8.14 (dd, 1H, *J_1_* = 9.5 Hz, *J_2_* = 2.5 Hz, H-6), 7.50–7.52 (m, 3H, H-4′, H-5′ and H-6′), 7.44–7.45 (m, 1H, H-2′), 2.40 (s, 3H, CH_3_); HRMS (TOF) calc. for C_16_H_11_ClN_3_O_2_ [M + H]^+^: 312.0534, found: 312.0536.

*7-Chloro-3-(3-chlorophenyl)quinoxaline-2-carbonitrile-1,4-dioxide *(**9r**). Yellow solid (45.8%); m.p.: 225–226 °C; IR (KBr): ν 3103, 2234, 1595, 1487, 1401, 1332, 1088, 987, 829 cm^−1^; ^1^H-NMR (CDCl_3_) δ 8.56–8.67 (m, 2H, H-5 and H-8), 7.88–7.94 (m, 1H, H-6), 7.74 (s, 1H, H-2′), 7.56–7.62 (m, 4H, H-6, H-4′, H-5′ and H-6′); HRMS (TOF) calc. for C_15_H_8_Cl_2_N_3_O_2 _[M + H]^+^: 331.9988, found: 331.9993.

*7-Chloro-3-(4-bromophenyl)quinoxaline-2-carbonitrile-1,4-dioxide *(**9s**). Yellow solid (49.4%); m.p.: 223–224 °C; IR (KBr): ν 3102, 2235, 1600, 1492, 1332, 1220, 1091, 989, 835 cm^−1^; ^1^H-NMR (DMSO-d_6_) δ 8.52-8.55 (m, 2H, H-5 and H-8), 8.15 (dd, 1H, *J_1_* = 9.5 Hz, *J_2_* = 2.5 Hz, H-6), 7.79–7.82 (m, 2H, H-3′ and H-5′), 7.46–7.50 (m, 2H, H-2′ and H-6′); HRMS (TOF) calc. for C_15_H_8_BrClN_3_O_2_ [M + H]^+^: 385.9483, found: 385.9489.

*7-Chloro-3-(4-nitrophenyl)quinoxaline-2-carbonitrile-1,4-dioxide *(**9t**). Yellow solid (47.9%); m.p.: 238–239 °C; IR (KBr): ν 3107, 2237, 1599, 1519, 1488, 1340, 1094, 987, 830 cm^−1^; ^1^H-NMR (DMSO-d_6_) δ 8.54–8.58 (m, 1H, H-5 and H-8), 8.48–8.50 (m, 2H, H-3′ and H-5′), 8.17–8.19 (m, 1H, H-6), 8.02–8.04 (m, 2H, H-2′ and H-6′); HRMS (TOF) calc. for C_15_H_8_ClN_4_O_4_ [M + H]^+^: 343.0229, found: 343.0234.

### 3.3. Pharmacology

The tested eleven human cancer cell lines (SMMC-7721, K562, KB, A549, PC-3, BEL-7402, HepG2, HL-60, NCI-H460, HCT-116 and CHP126) were purchased from the Cell Bank of China Science Academy (Shanghai, China). The above cells were cultured in RPMI-1640 (Invitrogen Corp., Carlsbad, CA, USA) medium with heat-inactivated 10% fetal bovine serum, penicillin (100 units/mL) and streptomycin (100 μg/mL) and incubated in normoxic atmosphere with 20% O_2_, 5% CO_2_ at 37 °C or in hypoxic atmosphere with 3% O_2_, 5% CO_2_ (established in a hypoxia incubator [Forma Scientific, Inc., Marietta, OH, USA] where N_2_ was used to compensate for the reduced O_2_ level).

#### 3.3.1. Cytotoxicity Assay [25]

Cancer cells were seeded in 96-well microtiter plates (4,000 cells/well), and were cultured in normoxia and hypoxia. The hypoxic cells were allowed to attach 1 day prior to the addition of these compounds (0–50 μM) in complete medium (to various final concentrations in 200 μL of complete medium) in replicates of 4 wells per condition. Plates were assayed at 72 h after initiation of drug exposure. Afterwards, 10 μL of stock 3-[4,5-dimethylthia-zol-2-yl]-2,5-diphenyltetrazolium bromide (MTT, Sigma) solution was added to each well (0.5 mg/mL) for another 4 h incubation (37 °C, 5% CO_2_). After 4 h incubation, 200 μL of DMSO was added to each well and optical density (OD) was read at 570 nm by Thermo Multiskan Spectrum (Thermo Electron Corporation). The IC_50_ values were calculated using the PrismPad computer program (GraphPad Software, Inc., San Diego, CA, USA) and were defined as concentration of drug causing 50% inhibition in absorbance compared with control (vehicle) cells.

#### 3.3.2. Flow Cytometry Analysis [25]

The SMMC-7721 cells were treated with **9h** and/or the general caspase inhibitor, z-VAD-fmk (R&D Systems, Inc., Minneapolis, MN, USA), **9h** (10 μM) alone for 24–48 h in 1% O_2_ or 20% O_2_ respectively; 9h (10 μM) +z-VAD-fmk (10.0 μM) for 48 h in hypoxia. Detection of apoptosis by FACS Calibur flow cytometer (Becton Dickinson, Lincoln Park, NJ, USA) was performed using the Propidium iodide (PI) apoptosis detection kit (BioVision, Mountain View, CA, USA).

#### 3.3.3. Western Blot Analysis [25]

Proteins of SMMC-7721 cells incubated with 10 μM **9h** both in hypoxia and normoxia were extracted in radio-immunoprecipitation assay buffer (150 mM NaCl, 50 mM Tris, 2 mM EGTA, 2 mM EDTA, 25 mM NaF, 25 mM glycerophosphate, 0.2% Triton X-100, 0.3% NONIDET P-40, 0.1 mM PMSF). Total protein concentrations of whole cell lysates were determined using BioRad BCA method (Pierce, Rockford, IL, USA). Equal amounts of protein sampled from whole cell lysates were subjected to electrophoresis on 8%–12% Tris-Glycine pre-cast gels (Novex, San Diego, CA, USA) and electroblottedonto Immobilon-P Transfer Membrane (Millipore Corporation, Billerica, MA, USA), and probed with primary antibodies and then incubated with a horseradish peroxidase (HRP) conjugated secondary antibodies. Proteins were visualized using enhanced chemiluminescent (ECL) Western Blotting detection reagents (Amersham Biosciences, Piscataway, NJ, USA).

## 4. Conclusions

In summary, a series of 3-aryl-2-quinoxalinecarbonitrile-1,4-di-*N*-oxide derivatives **9a**–**t** have been synthesized and evaluated for their hypoxic and normoxic cytotoxic activity. Many of these 3-aryl-2-quinoxalinecarbonitrile-1,4-di-*N*-oxide derivatives showed better hypoxic cytotoxic activity and higher hypoxic selectivity than that of TPZCN and TPZ against most tested cancer cell lines, in particular for the SMMC-7721, K562 and KB cell lines. The preliminary SAR study revealed that the 3-(3-chlorophenyl) moiety was favorable for hypoxic cytotoxicity and the 7-methyl or 7-methoxy moiety improved the hypoxic selectivity. Compound **9h** decreased the protein levels of procaspase-3 and induced the cleavage of PARP in hypoxia, which suggested it induces apoptosis in a caspase-dependent pathway. 
